# Cationic pillar[6]arene/ATP host–guest recognition: selectivity, inhibition of ATP hydrolysis, and application in multidrug resistance treatment†

**DOI:** 10.1039/c6sc00531d

**Published:** 2016-03-02

**Authors:** Guocan Yu, Jiong Zhou, Jie Shen, Guping Tang, Feihe Huang

**Affiliations:** a State Key Laboratory of Chemical Engineering, Center for Chemistry of High-Performance & Novel Materials, Department of Chemistry, Zhejiang University Hangzhou 310027 P. R. China fhuang@zju.edu.cn +86-571-8795-3189 +86-571-8795-3189; b Department of Chemistry, Institute of Chemical Biology and Pharmaceutical Chemistry, Zhejiang University Hangzhou 310027 P. R. China

## Abstract

Due to the differences in the cavity size of the hosts and the charge and length of the guests, a cationic water-soluble pillar[6]arene (WP6) selectively complexes with ATP to form a stable 1 : 1 inclusion complex WP6⊃ATP. This host–guest complexation was utilized to efficiently inhibit the hydrolysis of ATP, arising from the existence of the hydrophobic cavity of WP6. A folic acid functionalized diblock copolymer (FA-PEG-*b*-PAA) was employed to PEGylate WP6 to endow the polyion complex (PIC) micelles with specific targeting ability, preferentially delivering WP6 to folate receptor over-expressing KB cell. This host–guest complexation was further used to block the efflux pump to transport anticancer drugs out of cells by cutting off the energy source, which enhanced the efficacy of the cancer chemotherapy of DOX·HCl towards drug resistant MCF-7/ADR cell. This supramolecular method provides an extremely distinct strategy to potentially overcome multidrug resistance (MDR).

## Abstract 2

The efficacy of DOX·HCl against drug resistant human breast cancer cells is enhanced in the presence of a water-soluble pillar[6]arene (WP6) due to the inhibition of ATP hydrolysis upon formation of WP6⊃ATP in cells, providing a novel strategy to overcome multidrug resistance.

## Graphical abstract

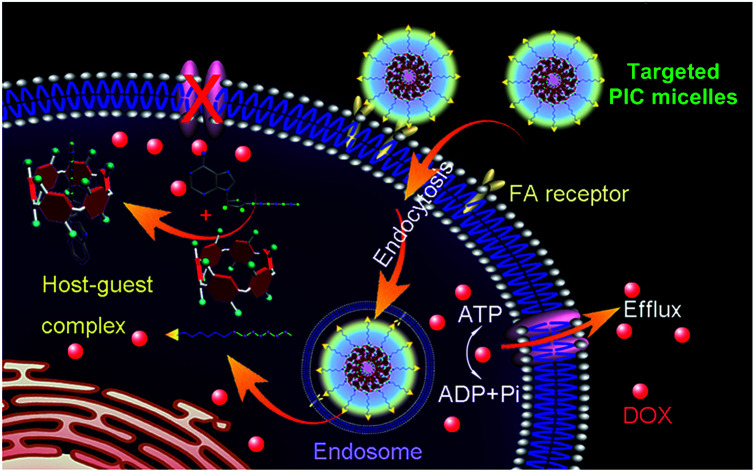

## Introduction

The recognition and sensing of biologically important nucleosides and nucleotides using artificial receptors has attracted considerable attention over the past decades, as they make up the fundamental building blocks of all life forms.^1^ Among all nucleotides, the selective recognition of adenosine 5′-triphosphate (ATP) is of particular interest, because it is a universal energy source and is the intracellular mediator in many biological processes.^2^ For example, P-glycoprotein (P-gp) functions by increasing the efflux of drugs out of the cell against a concentration gradient and reducing the rate of influx of drugs by utilizing the energy from ATP hydrolysis, thereby decreasing intracellular levels of the drugs below their level of effectiveness, a phenomenon that leads to multidrug resistance (MDR).^3^ In cells, ATP is enzymatically hydrolyzed to PPi (pyrophosphate) and adenosine 5′-monophosphate (AMP) or Pi (orthophosphate) and adenosine 5′-diphosphate (ADP). This imposes certain conditions on a receptor to have excellent selectivity between these anions, especially for ATP. Most known artificial receptors for the detection of ATP use complementary hydrogen bonding for their recognition, which is greatly limited in the aqueous medium arising from the competitive hydrogen bonding of the solvent.^4^ Therefore, it is an urgent issue to search for a novel receptor to ATP with excellent selectivity.

Analogous to crown ethers, cyclodextrins, calixarenes, cucurbiturils and other macrocycles,^5^ pillararenes,^6^ mainly including pillar[5]arenes and pillar[6]arenes, are a new kind of macrocycles composed of electron-donating 2,5-dialkoxybenzene units which are linked by methylene (–CH_2_–) bridges at their *para*-positions, forming a unique rigid pillar architecture. Due to their unique symmetrical structures and highly tunable functionalities, various interesting pillararene-based supramolecular systems, including liquid crystals,^7^ cyclic dimers,^8^ chemosensors,^9^ supramolecular polymers,^10^ drug delivery systems,^11^ metal–organic frameworks,^12^ transmembrane channels,^6*g*^ cell glue^13^ and theranostic rotaxane^6*j*^ have been constructed. Actually, highly tunable functionalities have endowed pillararenes with outstanding abilities to selectively bind different kinds of guests. It is known that the diameter of the internal cavity of pillar[5]arenes (∼4.7 Å) is smaller than that of pillar[6]arenes (∼6.7 Å).^14^ Therefore, pillar[6]arenes can complex with larger guest molecules to form inclusion complexes, such as azobenzene, 1,4-diazabicyclo[2.2.2]octane, tropylium tetrafluoroborate, adamantaneammonium, ATP and so on.^15^ For example, Diao *et al.* constructed an enzyme-responsive supramolecular system in water by using the recognition between an amphiphilic pillar[6]arene and ATP.^15*f*^

**Fig. 1:**
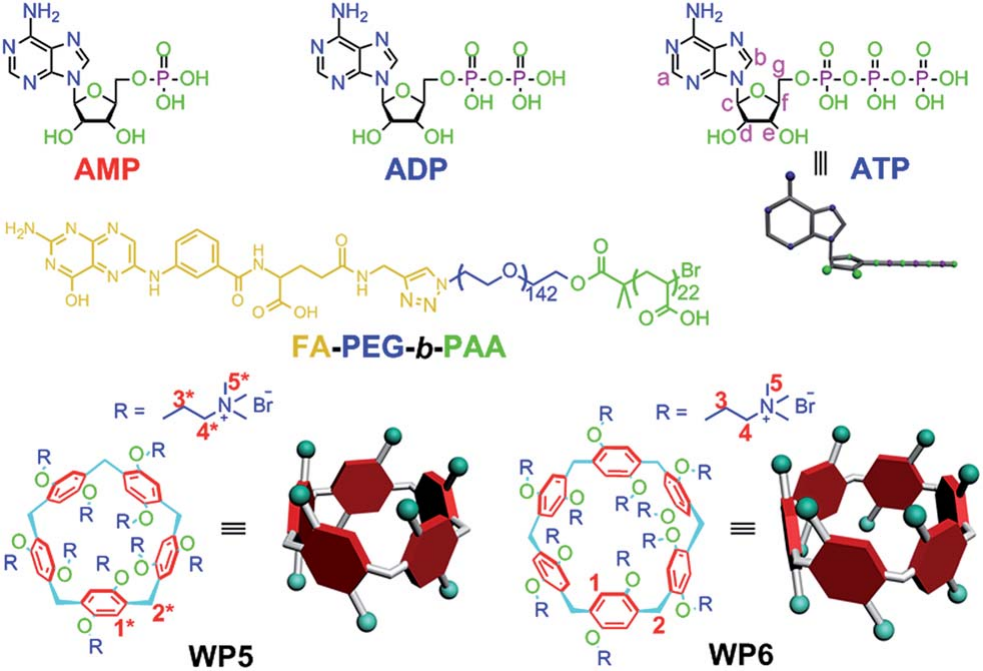
Chemical structures of AMP, ADP, ATP, WP5, WP6 and FA-PEG-*b*-PAA.

Herein, we investigate the host–guest complexation between cationic water soluble pillar[*n*]arenes (Fig. 1, *n* = 5 and 6, WP5 and WP6) and ribonucleotides (Fig. 1, AMP, ADP and ATP). Due to the differences in the cavity size of the hosts and the charge and length of the guests, WP6 bearing six trimethylammonium groups on both sizes selectively complex with ATP. This host–guest recognition motif is employed to efficiently inhibit the hydrolysis of ATP in the presence of alkaline phosphatase (CIAP). This can be potentially utilized to overcome MDR. PEGylation by a hydrophilic diblock copolymer poly(ethylene glycol)_142_-*block*-(polyacrylic acid)_22_ (Fig. 1, FA-PEG-*b*-PAA) endows the formed polyion complex (PIC) micelles with excellent targeting ability, preferentially delivering WP6 to folate receptor over-expressing cancer cells. The ATP-dependent drug efflux pump was abated efficiently due to the inhibition of ATP hydrolysis by the formation of a stable host–guest inclusion complex WP6⊃ATP. As a consequence, the efficacy of cancer chemotherapy of doxorubicin hydrochloride (DOX·HCl) was significantly improved towards drug resistant human breast cancer cells (MCF-7/ADR) in the presence of WP6.

## Results and discussion

**Fig. 2:**
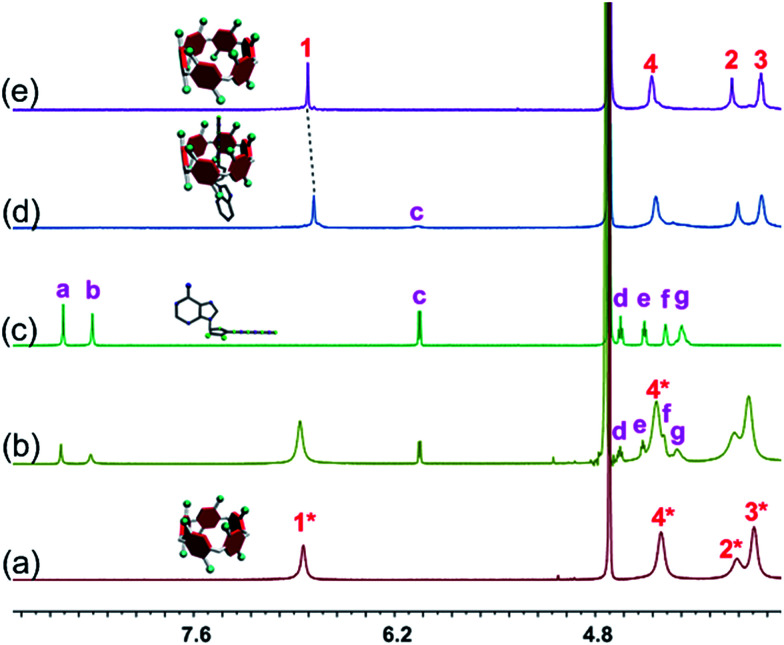
Partial ^1^H NMR spectra (400 MHz, D_2_O, 295 K): (a) WP5 (1.00 mM); (b) WP5 (1.00 mM) and ATP (1.00 mM); (c) ATP (1.00 mM); (d) WP6 (1.00 mM) and ATP (1.00 mM); (e) WP6 (1.00 mM).

The host–guest complexations between the pillararene hosts (WP5 and WP6) and guests (AMP, ADP and ATP) were first studied by ^1^H NMR spectroscopy. Compared with the free guest (AMP, ADP or ATP), negligible chemical shift changes were observed for the protons on these ribonucleotide guests in the presence of WP5 (Fig. 2b, S10 and S12†). The reason was that the cavity of WP5 was too small to encapsulate the adenosine group. On the other hand, almost no chemical shifts were observed for the protons on AMP (or ADP) in the presence of WP6 (ESI, Fig. S14 and S16†) either, suggesting that the complexations between WP6 and AMP and between WP6 and ADP were not strong enough to include the adenosine group into the cavity of WP6. However, the resonance peaks related to protons H_a_, H_b_, H_d_, H_e_, H_f_ and H_g_ of ATP disappeared, caused by a broadening effect after complexation upon addition of 1.0 equiv. of WP6 (Fig. 2d). The reason was that these protons were located within the cavity of WP6 and were shielded by the electron-rich cyclic structure by forming an inclusion complex between WP6 and ATP.^6*e*^ On the other hand, extensive broadening effect and upfield shifts were observed for the peak corresponding to proton H_c_ due to complexation dynamics.^14^ The results obtained from ^1^H NMR spectra demonstrated that WP6 could selectively complex with ATP, because the cavity size of WP6 was suitable for ATP and the binding affinity was strong enough to form an inclusion host–guest complex mainly through electrostatic interactions and π–π interactions between benzene rings and adenine.

^31^P NMR spectroscopy was also conducted to further verify the size-selective host–guest complexation between WP6 and ATP (ESI, Fig. S11, S13, S15, S17 and S18†). No changes corresponding to the phosphorus signals of AMP (or ADP) were observed upon addition of WP5 or WP6 (3 equiv.). This was also true for the case of WP5 and ATP. These phenomena were in good agreement with the results obtained from ^1^H NMR studies (Fig. 2, S10, S12, S14 and S16†). On the contrary, the peaks related to the resonances of α-ATP, β-ATP and γ-ATP all became broad and showed chemical shift changes upon addition of WP6 (ESI, Fig. S18†), indicating successful complexation. A possible reason was that ATP was located in the cavity of WP6 upon formation of an inclusion complex and shielded by its electron-rich cyclic structure.

Isothermal titration calorimetry (ITC) experiments were further performed to provide thermodynamic insight into the inclusion complexation between the pillar[*n*]arenes (*n* = 5, 6) and ribonucleotides. As shown in Table 1, the enthalpy and entropy changes were obtained (Δ*H*° > 0; *T*Δ*S*° > 0; |Δ*H*°| < |*T*Δ*S*°|), indicating that the complexations were driven by entropy changes. From comparison of the association constant (*K*_a_) values of these host–guest systems, we know that WP6⊃ATP exhibited the highest binding affinity, which further confirmed that WP6 selectively complexed with ATP. Furthermore, NOE correlation signals were observed between proton H_c_ on the ribose unit of ATP and proton H_1_ on the benzene rings of WP6 from the 2D NOESY NMR spectrum (ESI, Fig. S26†), indicating that ATP penetrated into the cavity of WP6 to form a [2]pseudorotaxane-type inclusion complex. Fig. S27† shows the energy-minimized structure of WP6⊃ATP with the guest molecule tightly wrapped with WP6. Noticeably, the anionic segment of ATP locates on the upper side of WP6 to successfully achieve multivalent electrostatic interactions with the cationic trimethylammonium groups. On the other hand, the repeating units of WP6 rotate around the methylene bridges, changing its appearance from the pillar structure to a conical structure due to the asymmetric complexation.^9*b*^ The ribose group threads into the cavity of WP6 and it is surrounded by the benzene rings. Furthermore, the largest part (adenine group) of the ATP guest is situated at the relatively larger side of the conic WP6. The information obtained from molecular modeling is in good agreement with the NMR results mentioned above. Moreover, fluorescence titration experiments were carried out to provide evidence for the interactions between WP6 and ATP. As shown in Fig. S25,† addition of ATP to a phosphate buffer solution of WP6 resulted in a substantial decrease in the intensity of the emission band at 325 nm caused by host–guest complexation between WP6 and ATP.

**Table 1:** Thermodynamic data, including association constants (*K*_a_), enthalpy changes (Δ*H*), and entropy changes (Δ*S*), obtained from ITC experiments for the complexes of WP5 (or WP6) with the guests (AMP, ADP or ATP)^a^

Host	Guest	10^−3^*K*_a_/M^−1^	Δ*H*/kcal mol^−1^	Δ*S*/cal mol^−1^ K^−1^
WP5	AMP	2.78 ± 0.35	85.9 ± 6.7	304
WP5	ADP	3.92 ± 0.43	64.3 ± 5.1	232
WP5	ATP	26.2 ± 3.30	8.99 ± 0.76	50.4
WP6	AMP	2.86 ± 0.23	42.8 ± 2.2	159
WP6	ADP	4.38 ± 0.43	16.9 ± 1.4	73.4
WP6	ATP	152 ± 16	4.58 ± 0.42	39.1

a Microcalorimetric titration experiments were conducted in PBS (pH = 7.4) at 298.15 K by titration of AMP (ADP or ATP) (2.00 mM, 10 μL per injection) into the solution of WP5 (or WP6) (0.100 mM).

From the above discussions, we can know that the adenosine group of the guest is included in the cavity of WP6 for WP6⊃ATP while this group is not in the cavity of WP6 for WP6⊃AMP and WP6⊃ADP. This should be ascribed to the differences in charge and length of these three guests. The length of the ATP is appropriate to maximize both multiple electrostatic interactions between the anionic segment of ATP and cationic ammonium units and π–π interactions between benzene rings and the adenine group, as shown by the energy-minimized structure of WP6⊃ATP (ESI, Fig. S27†).

More interestingly, the hydrolysis of ATP was inhibited efficiently in the presence of WP6, in sharp comparison with the case of free ATP (ESI, Fig. S28–S30†). As indicated by ^31^P NMR spectra, the hydrolysis rate of ATP slowed down efficiently by forming a stable host–guest inclusion complex WP6⊃ATP in the presence of CIAP (6 U mL^−1^) (ESI, Fig. S30†). The reason was that ATP was located in the hydrophobic cavity of WP6, preventing ATP from being hydrolyzed.^16^ The development of MDR to a variety of chemotherapeutic agents is one of the major challenges for efficient cancer treatment. One of the major causes of MDR is the over-expression of the drug efflux pump, the ATP-binding cassette superfamily membrane proteins, which transport anticancer drugs out of the cells and result in drug resistance by utilizing the energy from ATP hydrolysis.^17^ Among various ATP-binding cassette superfamily membrane proteins, P-gp known as the ATP-dependent drug efflux pump which is upregulated in the plasma membrane of all MDR cancer cells, is probably the best characterized ABC transporter.^18^ If the hydrolysis of ATP can be inhibited, the source of the chemical energy is cut off, resulting in the blocking of the efflux pump. On the other hand, the anticancer drugs can hardly be transported out of the cells through other energy-dependent approaches due to the shortage of energy, thus the efficacy of the cancer chemotherapy can be improved significantly.

In order to specifically block the cancer cells rather than normal cells, a folic acid functionalized hydrophilic diblock copolymer FA-PEG-*b*-PAA was chosen to PEGylate the cationic WP6. Here WP6 worked as a supramolecular cross-linker to interact with the negatively charged carboxylate segments of the diblock copolymer through electrostatic interactions in the buffer, forming stable polyion complex (PIC) micelles where WP6 molecules and the anionic carboxylates are in the core of the PIC micelles, and the PEG blocks are on the surfaces of the micelles.^19^ FA groups decorated on the surfaces of the PIC micelles endow these nanovehicles with the ability to specifically deliver WP6 to folate receptor (FR) over-expressing cancer cells, because FR is a well-known tumor-associated receptor that is over-expressed in many tumors, including those of the breast, lung, kidney, and brain (*e.g.*, HeLa and KB cell lines), relative to normal tissue. On the other hand, PEGylation could also be employed to reduce the cytotoxicity of the cationic WP6.

In order to monitor the *in vitro* cell accumulation of the ternary PIC micelles as well as to confirm targeting delivery of these nanocarriers to FR over-expressing cancer cells, DOX·HCl as a commonly used anticancer drug with strong fluorescence was loaded into the PIC micelles formed by FA-PEG-*b*-PAA and WP6. TEM was employed to reveal the morphology of the ternary self-assembly formed by FA-PEG-*b*-PAA, WP6 and DOX·HCl (charge ratio *r* = 1 : 1). As shown in Fig. S31a,† spherical aggregates were observed with diameters ranging from 100 to 170 nm, in good agreement with the results obtained from DLS (166 nm, ESI, Fig. S31b†). It should be emphasized that the DOX·HCl loaded ternary PIC micelles were quite stable in the buffer and showed no structural changes for several weeks.

**Fig. 3:**
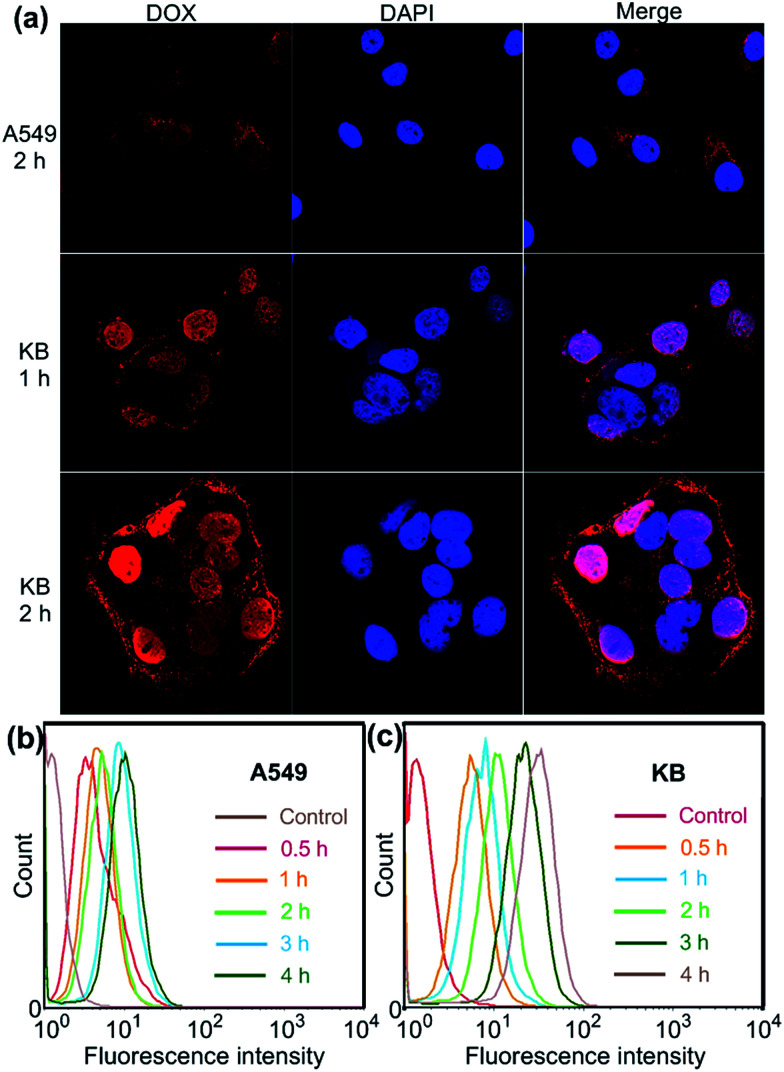
(a) Confocal laser scanning microscopy (CLSM) images of A549 and KB cells incubated with DOX·HCl (5.00 μg mL^−1^) loaded WP6/FA-PEG-*b*-PAA ternary PIC micelles. Flow cytometry showing increasing uptake of DOX·HCl (5.00 μg mL^−1^) loaded WP6/FA-PEG-*b*-PAA ternary PIC micelles over a 4 h period using A549 (b) and KB (c) cells.

Confocal laser scanning microscopic (CLSM) and flow cytometry investigations were conducted to verify whether the FA moieties decorated on the surface of the PIC micelles could guide these nanocarriers containing WP6 preferentially to FR over-expressing KB cells, rather than FR low-expressing A549 cells. As shown in Fig. 3a, KB cells exhibited strong intracellular DOX·HCl fluorescence after incubation with DOX·HCl (5.00 μg mL^−1^) loaded ternary PIC micelles for 1 h, and the fluorescence intensity became much stronger associated with extension of incubation time to 2 h. However, A549 cells showed weak fluorescence signal under the same experimental conditions (Fig. 3a). Moreover, red dots corresponding to the ternary PIC micelles affixed on the membrane of KB cells, indicating strong interactions between FA groups on the surfaces of the ternary PIC micelles and FRs on the cell membrane. Flow cytometry investigations demonstrated that KB cells always had faster uptake rate and higher intracellular accumulation than A549 cells (Fig. 3b and c). The mean fluorescence intensity was three times greater in KB cells than that in A549 cells (31.7 ± 0.93 *vs.* 10.6 ± 0.78) after incubation with the ternary PIC micelles for 4 h (ESI, Fig. S32†). These phenomena confirmed that the FA modified ternary PIC micelles could specifically enter the KB cells aided by the interaction of the FA moieties with FRs on the KB cells, possibly *via* receptor-mediated endocytosis.

**Fig. 4:**
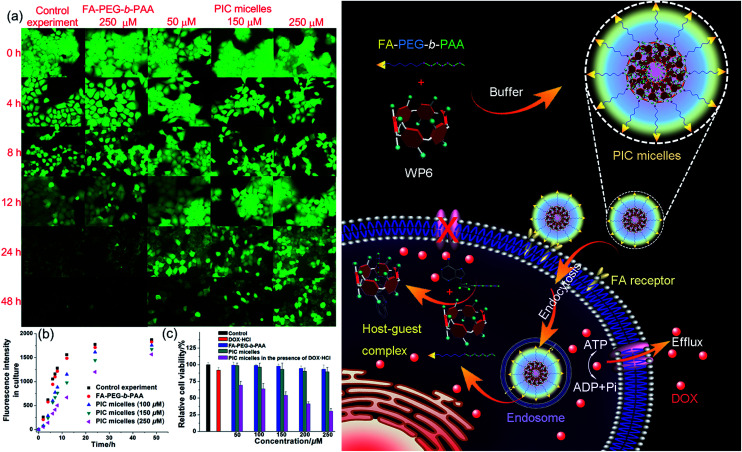
(a) Fluorescence images of the MCF-7/ADR cells stained with calcein-AM incubated without/with FA-PEG-*b*-PAA (250 μM), PIC micelles containing different amount of WP6. (b) Fluorescence intensity changes of the culture in the presence of FA-PEG-*b*-PAA or PIC micelles containing different amounts of WP6. (c) Cytotoxicity of DOX·HCl (25 μM), FA-PEG-*b*-PAA, PIC micelles, and DOX·HCl (25 μM) loaded PIC micelles with different concentrations of WP6 against MCF-7/ADR cells. Schematic illustration of the preparation of PIC micelles and possible mechanism to inhibit the efflux pump by forming a host–guest complex WP6⊃ATP in the cell.

Induced by the hydrophobicity, calcein acetoxymethyl ester (calcein-AM) can diffuse across the cell membranes, and be hydrolyzed to hydrophilic fluorescent calcein by esterase in the cytoplasm, so it can be employed as an excellent probe to test the operation of the efflux pumps in cell membranes by monitoring the fluorescence changes of the cell culture.^20^ Fluorescence images provided visual colour changes of the cells stained with calcein in the presence and absence of WP6 by culturing the cell for 48 h. As shown in Fig. 4a, the colour of the cells in the absence of WP6 became weaker with the extension of culture time, indicating that calcein had a fast and profound release. In a parallel study by fluorescence microscopic observation, a similar phenomenon was observed for cells under the same incubation conditions in the presence of FA-PEG-*b*-PAA. While the fluorescence intensity of the cells changes much slower in the presence of WP6 owning to the inhibition of ATP hydrolysis by forming WP6⊃ATP. Fig. 4b shows the release profile of calcein from the drug resistant MCF-7/ADR cells. Regardless of the presence of WP6, the release rate and the total release of calcein from cells were much higher than those in the presence of WP6 by determining the fluorescence intensity of the cell culture. Accompanied with the enhancement of the concentration of WP6 from 100 to 250 μM, the release rate and the total release of calcein from cells slowed down efficiently caused by the inhibition of ATP hydrolysis (Fig. 4b). The ATP-dependent calcein release underlined the significant role of WP6 in the influence of the efflux pump by cutting off the energy source from ATP hydrolysis due to the formation of the host–guest inclusion complex.

The efficacy of the cancer chemotherapy of DOX·HCl in the presence and absence of WP6 against MCF-7/ADR cell lines was carried out using 3-(4′,5′-dimethylthiazol-2′-yl)-2,5-diphenyltetrazolium bromide (MTT) assay. As control experiments, the viabilities of MCF-7/ADR cells cultured with the diblock polymer FA-PEG-*b*-PAA or the PIC micelles were investigated, which showed minor cytotoxicity with concentrations ranging from 50 to 250 μM (Fig. 4c). On the other hand, the relative cell viability was 91.8% by culturing the cells with DOX·HCl (25 μM) (Fig. 4c). In stark contrast, the viability of MCF-7/ADR cells decreased from 69.2% to 30.3% accompanied with the increase of WP6 from 50 to 250 μM in the presence of DOX·HCl at the same concentration (25 μM). The reason was that the source of the chemical energy was cut off by forming the stable inclusion complex WP6⊃ATP, resulting in the blocking of the efflux pump and other energy-dependent approaches to transport DOX·HCl out of cells. Therefore, the intracellular concentration of DOX·HCl was higher in the presence of WP6 than those in the absence of WP6, resulting in the reduction of the corresponding relative cell viability, which indicated that the efficacy of the cancer chemotherapy DOX·HCl was improved significantly.

## Conclusions

In summary, a cationic water-soluble pillar[6]arene (WP6) selectively complexed with ATP to form a stable 1 : 1 inclusion complex WP6⊃ATP mainly driven by entropy change. As a result, the hydrolysis of ATP was efficiently inhibited in the presence of alkaline phosphatase due to the existence of a hydrophobic cavity of WP6. A folic acid ended diblock polymer FA-PEG-*b*-PAA was employed to PEGylate the cationic pillar[6]arene WP6 to obtain PIC micelles in buffer, endowing them with specific targeting ability to deliver WP6 to folate receptor over-expressing cancer cells. The ATP-dependent efflux pump was blocked by cutting off the energy source from ATP hydrolysis due to the formation of the host–guest inclusion complex. Furthermore, MTT assay demonstrated that the efficacy of the anticancer drug DOX·HCl was improved effectively in the presence of PIC micelles. The present results pave a way to develop novel therapeutic agents, implying that supramolecular chemistry may be engineered into promising vehicles to overcome multidrug resistance in cancer therapy. More detailed biologic investigations need to be carried out to figure out the deeper-level mechanism of the MDR treatment.

## Supplementary Material

SC-007-C6SC00531D-s001
