# Supplementary material for: Cationic pillar[6]arene/ATP host–guest recognition: selectivity, inhibition of ATP hydrolysis, and application in multidrug resistance treatment
Source: Chem Sci. 2016 Mar 2;7(7):4073–8. doi: 10.1039/c6sc00531d (PMC6013913; doi:10.1039/c6sc00531d)
Supplement: SC-007-C6SC00531D-s001 [file SC-007-C6SC00531D-s001.pdf]

Electronic Supplementary Material (ESI) for Chemical Science.
This journal is © The Royal Society of Chemistry 2016

Cationic pillar[6]arene/ATP host−guest recognition: selectivity, inhibitation of ATP hydrolysis, and application in multidrug resistance treatment

Guocan Yu,[a,‡] Jiong Zhou,[a,‡] Jie Shen,[b] Guping Tang[b] and Feihe Huang*,[a]

[a] *State Key Laboratory of Chemical Engineering, Center for Chemistry of High-Performance & Novel Materials, Department of Chemistry, Zhejiang University, Hangzhou 310027, P. R. China; Fax and Tel: +86-571-8795-3189; Email: fhuang@zju.edu.cn*

[b] *Department of Chemistry, Institute of Chemical Biology and Pharmaceutical Chemistry, Zhejiang University, Hangzhou 310027, P. R. China.*

‡ These authors contributed equally to this work.

Electronic Supplementary Information

1. *Materials and methods*
2. *Syntheses of **WP6** and **FA-PEG-b-PAA***
3. *Host–guest interactions between **WP5** (or**WP6**) and ribonucleotides (AMP, ADP or ATP,)*
4. *Host-induced inhibition of ATP hydrolysis*
5. *Characterization of DOX·HCl-loaded PIC micelles*
6. *In vitro cell accumulation by confocal laser scanning microscopy (CLSM) and flow cytometry*

1. Materials and methods

Adenosine 5'-monophosphate (AMP), adenosine 5'-diphosphate (ADP) and adenosine 5’-triphosphate (ATP) were purchased from Aladdin (Shanghai, China). Solvents were either employed as purchased or dried according to procedures described in the literature. ^{1}H NMR and ^{13}C NMR spectra were recorded on a Bruker Avance Ⅲ-400 spectrometry. 2D NOESY NMR spectrum was collected on a Bruker Avance DMX-500 spectrometer with internal standard TMS. Low-resolution electrospray ionization (LRESI) mass spectra were obtained on a Bruker Esquire 3000 plus mass spectrometer (Bruker-Franzen Analytik GmbH Bremen, Germany) equipped with an ESI interface and an ion trap analyzer. HRMS were obtained on a WATERS GCT Premier mass spectrometer. The melting points were collected on a SHPSIC WRS-2 automatic melting point apparatus. MALDI-TOF spectrometry was performed on a 4700 MALDI-TOF (Applied Biosystems, U.S.A). The energy-minimized structure was calculated using the GAUSSIAN 03 software based on the arithmetic method PM3. The fluorescence experiments were conducted on a RF-5301 spectrofluorophotometer (Shimadzu Corporation, Japan). Transmission electron microscopy (TEM) investigations were carried out on a HT-7700 instrument. The ITC experiment was performed on a VP-ITC micro-calorimeter (Microcal, USA).

Preparation of DOX·HCl Loaded WP6/FA-PEG-*b*-PAA Ternary PIC Micelles. For the preparation of DOX·HCl loaded **WP6**/**FA-PEG-*b*-PAA** ternary PIC micelles, **WP6** (49.9 mg, 18.3 mmol) and DOX·HCl (100 μg) were first dissolved in water (0.5 mL). Then, the mixture was dropwisely added to a solution of **FA-PEG-*b*-PAA** (89.7 mg in 1.5 mL PBS) under stirring and sonication. The charge ratio r = 22 $[\textbf{FA-PEG-}\textbf{\textit{b}}\textbf{-PAA}]^{22-}/(12[\textbf{WP6}]^{12+} + [\text{DOX·HCl}]^{+})$ between **FA-PEG-*b*-PAA** and the cationic compounds (**WP6** and DOX·HCl) was 1:1.

Transmission Electron Microscopy (TEM) and Dynamic Light Scattering (DLS) Studies. The morphologies of DOX·HCl loaded **WP6/FA-PEG-*b*-PAA** ternary PIC micelles were revealed by TEM. The ternary micelles (the concentration of the polymer was 0.75 mg mL^{-1}) were prepared first in water. TEM samples were prepared by drop-

coating the solution on a carbon-coated copper grid. TEM experiments were performed on a HT-7700 instrument. The corresponding solution was left to stand overnight and the insoluble precipitate was eliminated by using a microporous membrane before being used for DLS tests. Dynamic light scattering (DLS) measurements were carried out using a 200 mW polarized laser source Nd:YAG (λ = 532 nm). The polarized scattered light was collected at 90° in a self-beating mode with a Hamamatsu R942/02 photomultiplier. The signals were sent to a Malvern 4700 submicrometer particle analyzer system.

Cell Culture. A549, KB and MCF-7/ADR cells were cultured in Dulbecco's modified Eagle's medium (DMEM) containing 10% fetal bovine serum (FBS) and 1% penicillin/streptomycin. Cells grew as a monolayer and were detached upon confluence using trypsin (0.5% *w*/*v* in PBS). The cells were harvested from cell culture medium by incubating in the trypsin solution for 5 min. The cells were centrifuged, and the supernatant was discarded. A 3.00 mL portion of serum-supplemented DMEM was added to neutralize any residual trypsin. The cells were resuspended in serum-supplemented DMEM at a concentration of 1.00×10^4 cells/mL. Cells were cultured at 37 °C and 5% CO_2.

***In Vitro* Cell Accumulation of the Ternary PIC Micelles Determined by Flow Cytometry.** Cellular uptake of DOX·HCl-loaded **WP6/FA-PEG-*b*-PAA** ternary PIC micelles was measured by flow cytometry. A549 and KB cells were seeded at a density of 3.00×10^5 cells/well in 12-well cell culture plates. The cells were left to grow for 24 h in DMEM media containing 10% FBS at 37 ^{o}C in 5% CO_2 atmosphere. After 24 h, DOX·HCl (5.00 μg/mL) loaded **WP6/FA-PEG-*b*-PAA** ternary PIC micelles were added to the wells and the cells were incubated for 30 min, 1 h, 2 h, 3 h, and 4 h, respectively. Following incubation, cells were rinsed twice with PBS to remove residual ternary PIC micelles. Cells were harvested by trypsinization and resuspended in 500 μL of PBS for flow cytometry analysis using the FACS Calibur flow cytometer (BD Facsealibur). Data shown are the mean fluorescent signal for 1.00×10^4 cells. Cells that were not treated with the ternary PIC micelles were used as a control. Data was analyzed using the FlowJo software.

***In Vitro* Cell Accumulation of the DOX·HCl Ternary PIC Micelles Determined by Confocal Laser Scanning Microscopy (CLSM).** A549 and KB cells were treated with DOX·HCl (5.00 μg/mL) loaded **WP6/FA-PEG-*b*-PAA** ternary PIC micelles in the culture medium at 37 °C for 2 h. The cells were washed three times with PBS and fixed with fresh 4.0% formaldehyde at room temperature for 15 min. After washing with PBS, the cells were stained with DAPI (1 μg/mL) for 15 min. The images were taken using a LSM-510 confocal laser scanning microscope (Zeiss, Germany) (100 × oil objective, 405/488 nm excitation).

Fluorescence Microscopy Experiments. MCF-7/ADR cells were seeded in a 96-well cell culture plate at a density of 1.00×10^4 cells per well and were allowed to attach for 24 h. To load calcein, firstly, MCF-7/ADR cells were incubated with its hydrophobic precursor, calcein acetoxymethyl ester (calcein-AM) (1 mg/mL stock solution in DMSO), at the concentration of 20 μg/mL in DMEM. After incubation at 37 °C for 30 min, cells were washed twice with PBS. **FA-PEG-*b*-PAA** (250 μM) or PIC ternary micelles containing different amounts of **WP6** were added. The control experiments were conducted in the absence of **FA-PEG-*b*-PAA** and **WP6**. The medium was removed for fluorescence determination. The cells were washed with PBS and then visualized under a fluorescence microscope (Nikon ECLIPSE Ti).

Evaluation of Cytotoxicity. The cytotoxicity of **FA-PEG-*b*-PAA**, PIC micelles, free DOX·HCl (25 μM) and DOX·HCl (25 μM) loaded PIC micelles containing different amounts of **WP6** against MCF-7/ADR cells were determined by 3-(4',5'-dimethylthiazol-2'-yl)-2,5-diphenyl tetrazolium bromide (MTT) assay in a 96-well cell culture plate. All solutions were sterilized by filtration with a 0.22 μm filter before tests. MCF-7/ADR cells were seeded at a density of 1.00×10^4 cells/well in a 96-well plate, and incubated for 24 h for attachment. Cells were then incubated with fresh serum-supplemented DMEM without/with **FA-PEG-*b*-PAA**, PIC micelles, DOX·HCl and PIC micelles at various concentrations in the presence of DOX·HCl (25 μM) for 4 h. Then 20 μL of a MTT solution (5.00 mg/mL) were added to each well. After 4 h of incubation at 37 °C, the

MTT solution was removed, and the insoluble formazan crystals that formed were dissolved in 100 μL of dimethylsulfoxide. The absorbance of the formazan product was measured at 570 nm using a spectrophotometer (Bio-Rad Model 680). Untreated cells in media were used as a control. All experiments were carried out with four replicates.

*2. Syntheses of **WP6** and **FA-PEG-b-PAA***

*2.1 Synthesis of **WP6***

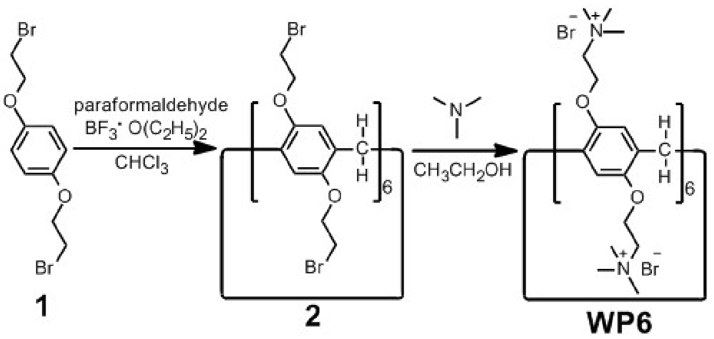

Scheme S1. Synthetic route to **WP6**.

Synthesis of **2**: To a solution of **1** (16.1 g, 50.0 mmol) in chloroform (300 mL), paraformaldehyde (3.00 g, 100 mmol) was added under nitrogen atmosphere. Then boron trifluoride diethyl etherate (10 mL) was added to the solution and the mixture was stirred at room temperature for 3 h. Water (50 mL) was added to quench the reaction. The mixture was filtered and the solvent was removed. The residue was dissolved in dichloromethane. The organic layer was dried over anhydrous Na_2SO_4 and evaporated to afford the crude product, which was isolated by flash column chromatography using ethyl acetate/petroleum ether (1:5) to give **2** as a white solid (2.10 g, 12%), mp: 81.6–82.9 °C. The ^1H NMR spectrum of **2** is shown in Fig. S1. ^1H NMR (400 MHz, $CDCl_3$, room temperature) δ (ppm): 6.78 (s, 12H), 4.16 (t, J = 4.0 Hz, 24H), 3.87 (s, 12H), 3.55 (t, J = 4.0 Hz, 24H). The ^{13}C NMR spectrum of **2** is shown in Fig. S2. ^{13}C NMR (100 MHz, $CDCl_3$, room temperature) δ (ppm): 150.2, 128.6, 115.9, 69.0, 30.7, 30.3. LRESIMS is shown in Fig. S3: m/z 2015.4 $[M + H]^+$ (100%). HRESIMS: m/z calcd for $[M + H]^+$ $C_{66}H_{73}Br_{12}O_{12}$, 2016.5135, found 2016.5178, error 2.1 ppm.

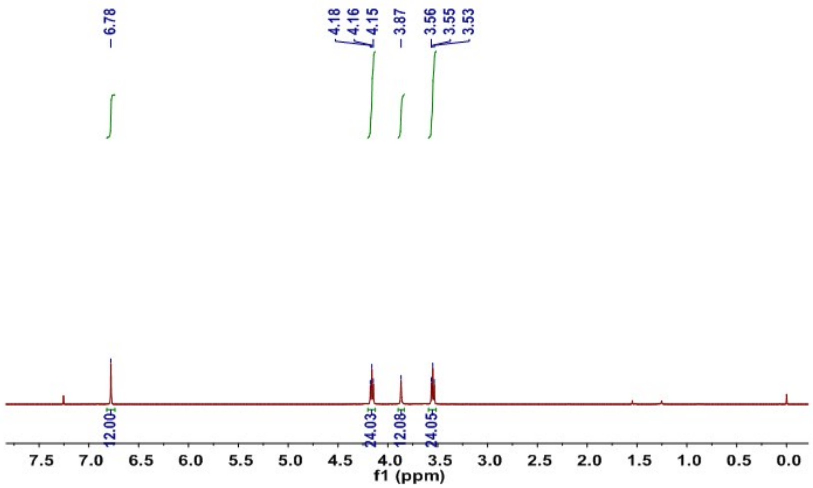

Fig. S1 ^{1}H NMR spectrum (400 MHz, chloroform-*d*, room temperature) of **2**.

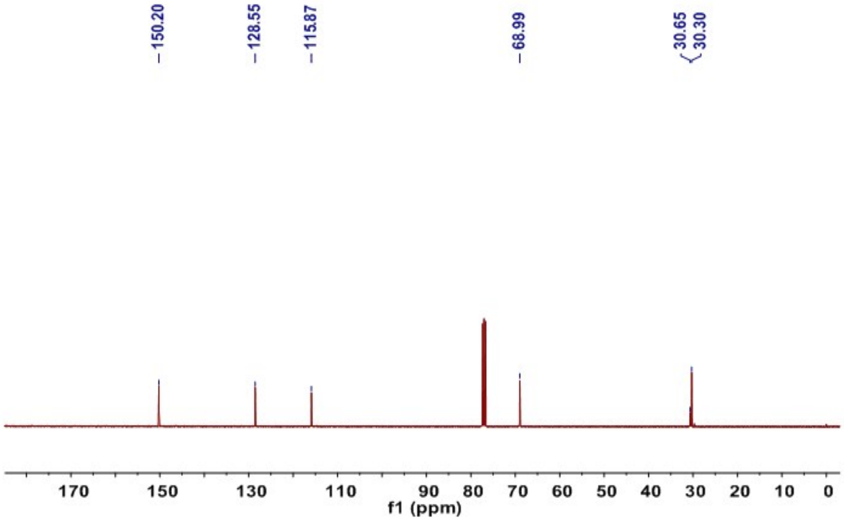

Fig. S2 ^{13}C NMR spectrum (100 MHz, chloroform-*d*, room temperature) of **2**.

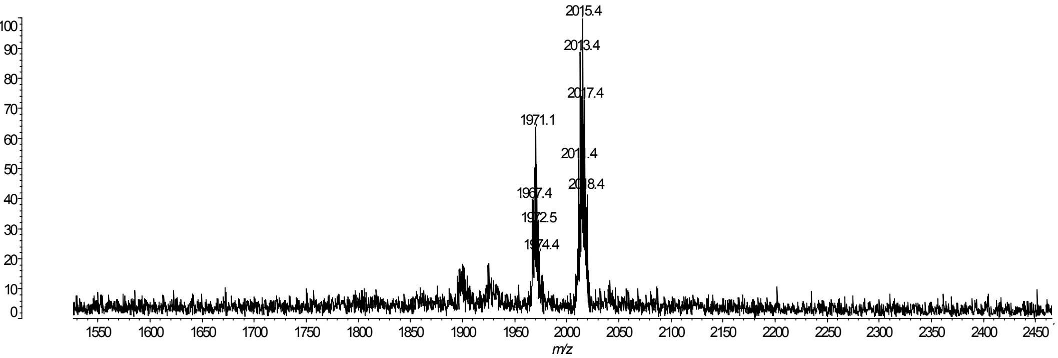

Fig. S3 MALDI-TOF mass spectrum of **2**. Assignment of the main peak: *m*/*z* 2015.4 $[M+H]^{+}$ (100%).

Synthesis of **WP6**: Compound **2** (1.00 g, 0.595 mmol) and trimethylamine (33% in ethanol, 6.43 mL) were added to ethanol (50 mL). The solution was refluxed overnight. The solvent was removed by evaporation. Deionized water (20 mL) was added. After filtration, a clear solution was got. Then the water was removed by evaporation to afford **WP6** as a colorless oil (1.28 g, 95 %). The ^{1}H NMR spectrum of **WP6** is shown in Fig. S4. ^{1}H NMR (400 MHz, D_2O, room temperature) δ (ppm): 6.85 (s, 12H), 4.36 (t, J = 4.0 Hz, 24H), 3.83 (s, 12H), 3.71 (t, J = 4.0 Hz, 24H), 3.12 (s, 108H). The ^{13}C NMR spectrum of **WP6** is shown in Fig. S5. ^{13}C NMR (125 MHz, D_2O, room temperature) δ (ppm): 149.32, 129.87, 116.44, 99.99, 64.86, 63.41, and 54.00. LRESIMS is shown in Fig. S6: m/z 374.3 $[M - 6Br]^{6+}$ (100%). HRESIMS: m/z calcd for $[M - 3Br]^{3+}$ $C_{102}H_{180}Br_9N_{12}O_{12}$, 828.5867, found 828.5886, error 2.3 ppm.

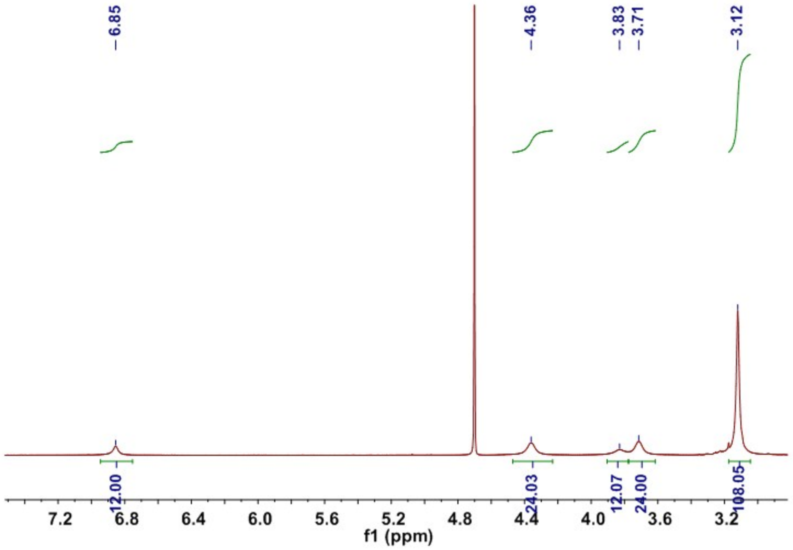

Fig. S4 ^{1}H NMR spectrum (400 MHz, D_2O, room temperature) of **WP6**.

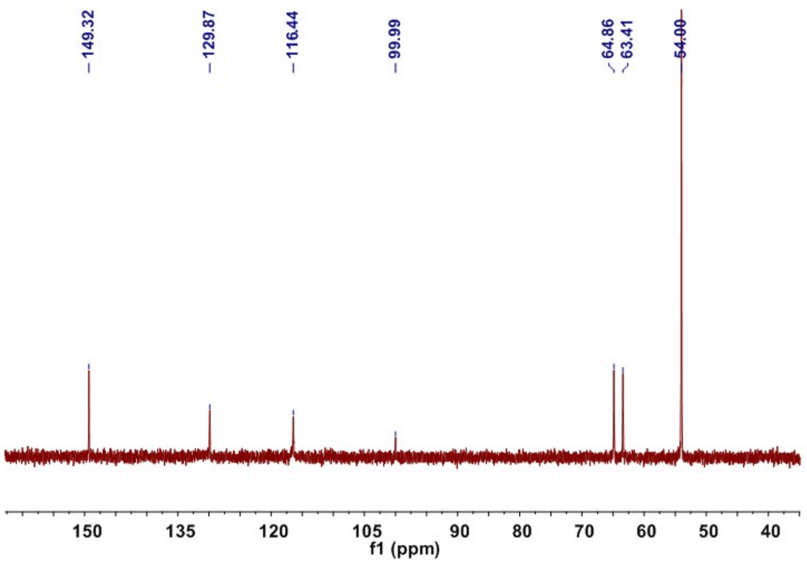

Fig. S5 ^{13}C NMR spectrum (100 MHz, D_2O, room temperature) of **WP6**.

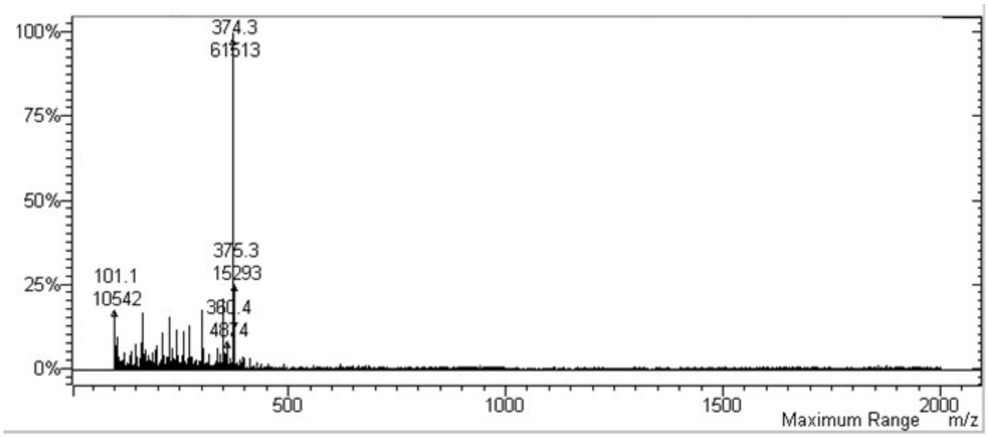

Fig. S6 Electrospray ionization mass spectrum of **WP6**. Assignment of the main peak: *m*/*z* 374.3 $[M - 6Br]^{6+}$ (100%).

*2.2 Synthesis of **FA-PEG-b-PAA***

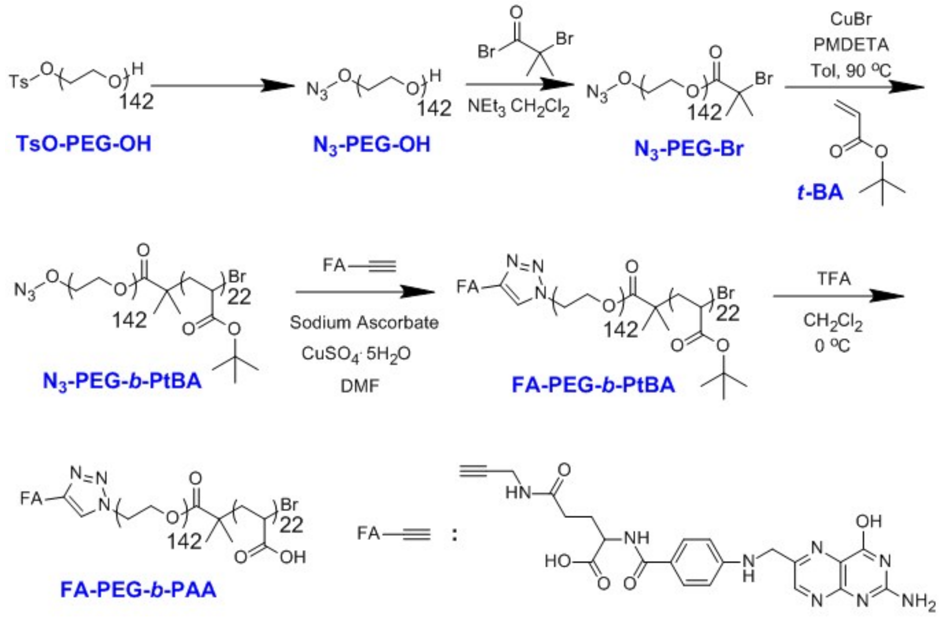

Scheme S2. Synthetic route to **FA-PEG-*b*-PAA**.

Synthesis of **N_3-PEG-OH**: A mixture of **TsO-PEG-OH** (6.64 g, 1.00 mmol) and NaN_3 (1.63 g, 25.0 mmol) was heated in *N,N'*-dimethylformamide (30.0 mL) at 90 °C for 12 h. The reaction mixture was diluted with dichloromethane (500 mL) and washed with water (3 × 200 mL). The organic phase was dried over magnesium sulfate and filtered. The solvent was removed under reduced pressure to afford **N_3-PEG-OH** as a white solid (5.09 g, 78%). Molecular weights and compositions of **N_3-PEG-OH** were determined by GPC (Fig. S7) and ^{1}H NMR spectroscopy (Fig. S8b).

Synthesis of **N_3-PEG-Br**: Triethylamine (2.02 g, 20.0 mmol) was added dropwisely to a solution of **2** (3.22 g, 0.500 mmol) and α-bromoisobutyryl bromide (1.15 g, 5.00 mmol) in dry THF (50 mL) at 0 °C. After warming up to room temperature, the mixture was further stirred for 12 h. After removal of insoluble salts by filtration, the solvent was removed on a rotary evaporator. The residues were dissolved in THF and precipitated into an excess of cold diethyl ether. The above dissolution-precipitation cycle was repeated three times. The final product was dried in a vacuum oven overnight at room temperature, yielding a white viscous solid (2.83 g, yield: 86%). Molecular weights and compositions of **N_3-PEG-Br** were determined by GPC (Fig. S7) and ^{1}H NMR spectroscopy (Fig. S8c).

Synthesis of **N_3-PEG-*b*-PtBA**: **N_3-PEG-Br** here was used as the ATRP macroinitiator. *t*-BA (7.57 mL, 52.2 mmol), **N_3-PEG-Br** (2.80 g, 0.435 mmol) and PMDETA (0.183 mL, 0.870 mmol) were charged into an ampoule tube, to which toluene (20.0 mL) was added to dissolve the monomer and initiator. After two cycles of freeze-pump-thaw to thoroughly remove oxygen, CuBr (61.8 mg, 0.435 mmol) was added into the tube under a nitrogen atmosphere, and the tube was sealed in vacuo. The polymerization was carried out at 90 ^oC for 80 min and then quenched by cooling in liquid nitrogen. After warming up to room temperature, the solution was diluted with THF and passed through a short basic Al_2O_3 column to remove the catalyst. The polymer was precipitated from cold petroleum ether thrice, and dried in vacuo to afford **N_3-PEG-*b*-PtBA** as a white power with the yield of 63%. Molecular weights and compositions of the block copolymers were determined by GPC (Fig. S7) and ^1H NMR spectroscopy (Fig. S8d).

Synthesis of **FA-PEG-*b*-PtBA**: Acetylated folic acid (FA-C≡C)[S1] (0.143 g, 0.300 mmol), copper sulfate pentahydrate (6.00 mg, 0.0240 mmol) and sodium ascorbate (22.5 mg, 0.160 mmol) were added to a solution of **N_3-PEG-*b*-PtBA** (1.89 g, 0.200 mmol) in *N,N'*-dimethylformamide (30.0 mL). The mixture was heated in a three-necked flask under nitrogen atmosphere at 90 °C for 24 h. The solvent was removed on a rotary evaporator, and the residue was diluted with dichloromethane (250 mL). The excess insoluble FA-C≡C was removed by filtration. The organic phase was dried over magnesium sulfate and filtered. The solvent was removed under reduced pressure to afford **FA-PEG-*b*-PtBA** as a light yellow solid with the yield of 87% (Fig. S8e).

Synthesis of **FA-PEG-*b*-PAA**: To a solution of **FA-PEG-*b*-PtBA** (1.49 g, 0.150 mmol) in dichloromethane (200 mL), trifluoroacetic acid (1.85 mL, 1.03 mmol) was added dropwise to the solution at 0 ^oC and the mixture was further stirred for 4 h. The organic solvent was removed under reduced pressure. The residue was washed with diethyl ether (2 × 50 mL) and dried in vacuo to afford **FA-PEG-*b*-PAA** as a light yellow solid with the yield of 86% (Fig. S8f).

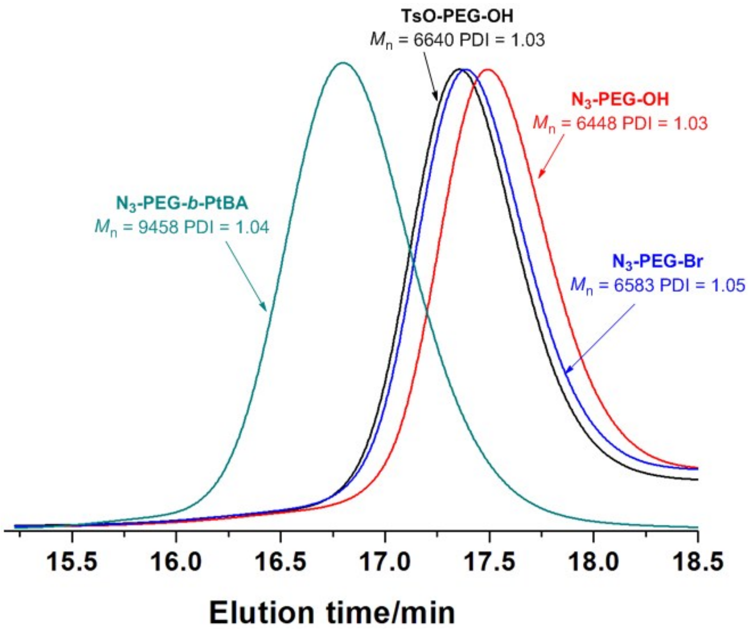

Fig. S7 GPC curves of polymers **TsO-PEG-OH**, **N_3-PEG-OH**, **N_3-PEG-Br** and **N_3-PEG-*b*-PtBA**.

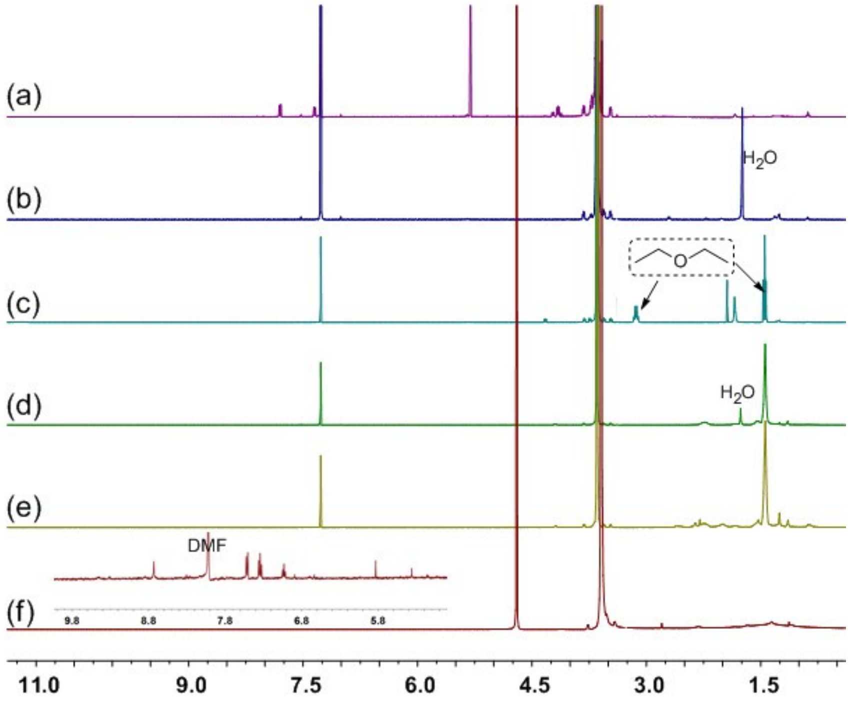

Fig. S8 ^{1}H NMR spectra: (a) **TsO-PEG-OH** (400 MHz, chloroform-*d*, room temperature); (b) **N_3-PEG-OH** (400 MHz, chloroform-*d*, room temperature); (c) **N_3-PEG-Br** (400 MHz, chloroform-*d*, room temperature); (d) **N_3-PEG-*b*-PtBA** (400 MHz, chloroform-*d*, room temperature); (e) **FA-PEG-*b*-PtBA** (400 MHz, chloroform-*d*, room temperature); (f) **FA-PEG-*b*-PAA** (400 MHz, D_2O, room temperature).

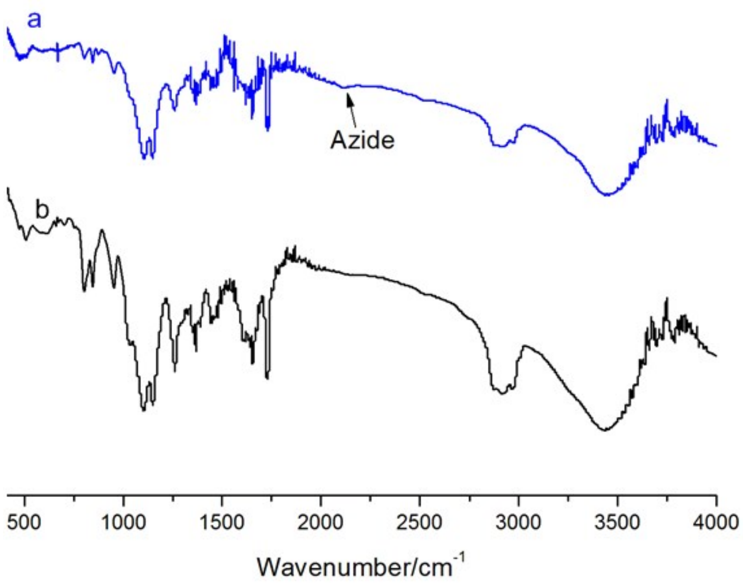

Fig. S9 FT-IR spectra of (a) **N_3-PEG-*b*-PtBA** and (b) **FA-PEG-*b*-PtBA**.

*3. Host–guest interactions between **WP5** (or**WP6**) and ribonucleotides (AMP, ADP or ATP)*

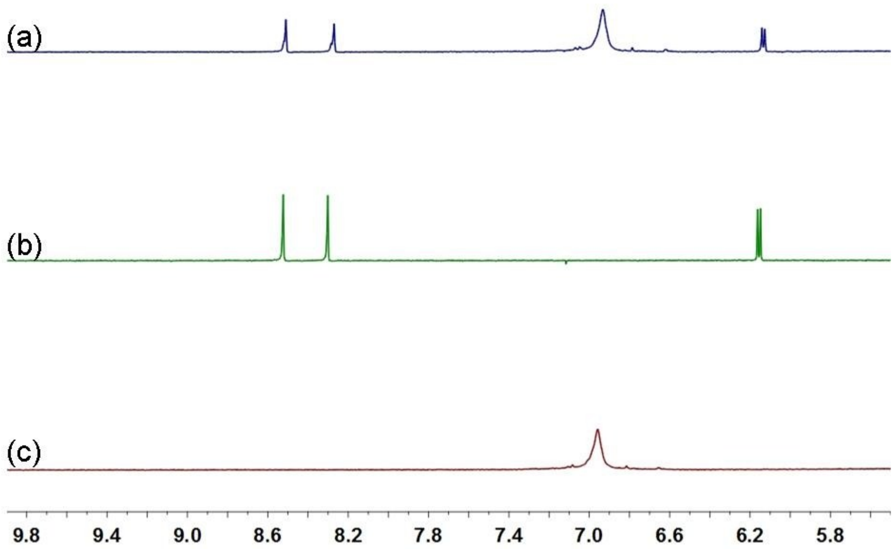

Fig. S10 Partial ^{1}H NMR spectra (400 MHz, D_2O, 295 K): (a) AMP (1.00 mM); (b) **WP5** (1.00 mM) and AMP (1.00 mM); (c) **WP5** (1.00 mM).

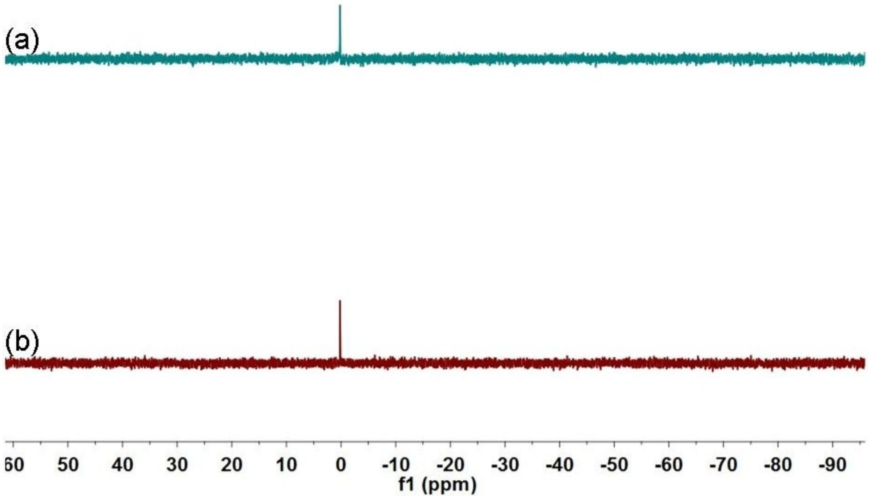

Fig. S11 ^{31}P NMR spectra of (a) AMP (1.00 mM), (b) **WP5** (1.00 mM) and AMP (1.00 mM).

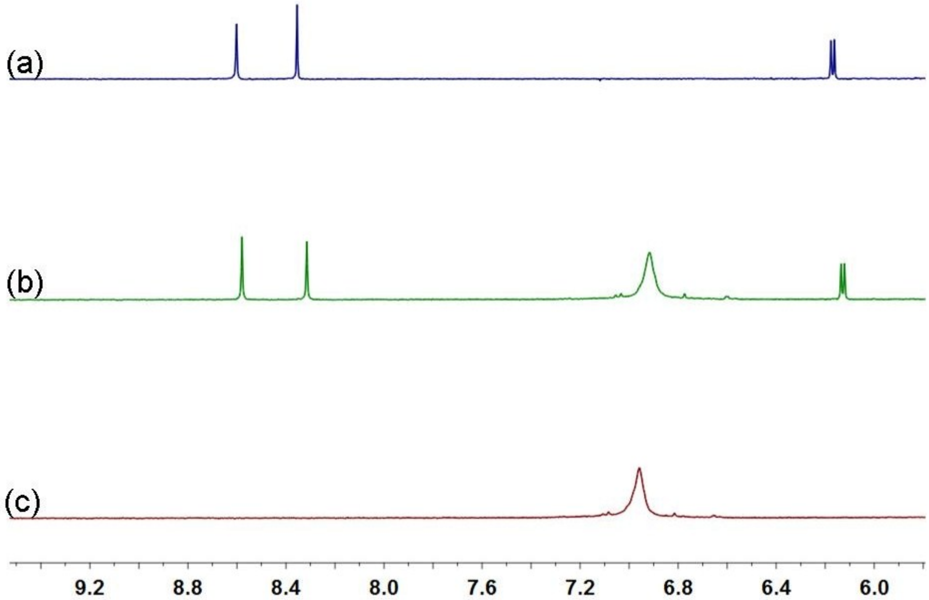

Fig. S12 Partial ^{1}H NMR spectra (400 MHz, D_2O, 295 K): (a) ADP (1.00 mM); (b) **WP5** (1.00 mM) and ADP (1.00 mM); (c) **WP5** (1.00 mM).

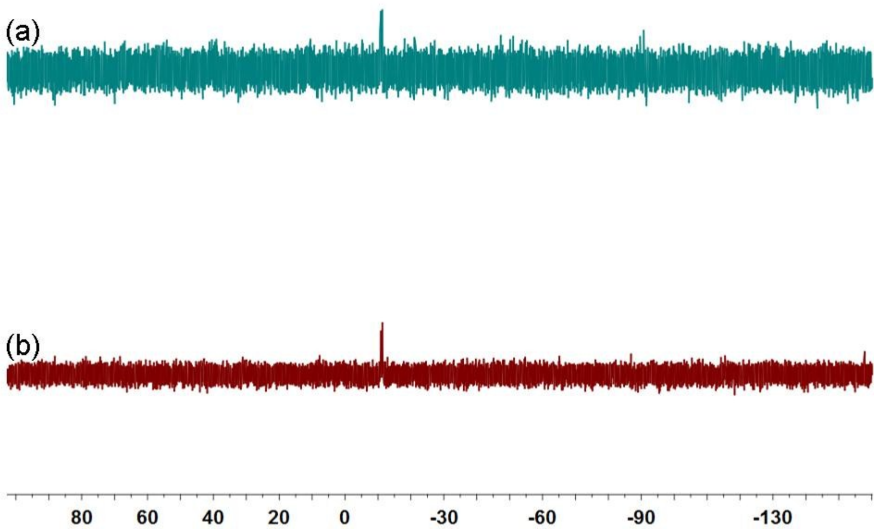

Fig. S13 ^{31}P NMR spectra of (a) ADP (1.00 mM), (b) **WP5** (1.00 mM) and ADP (1.00 mM).

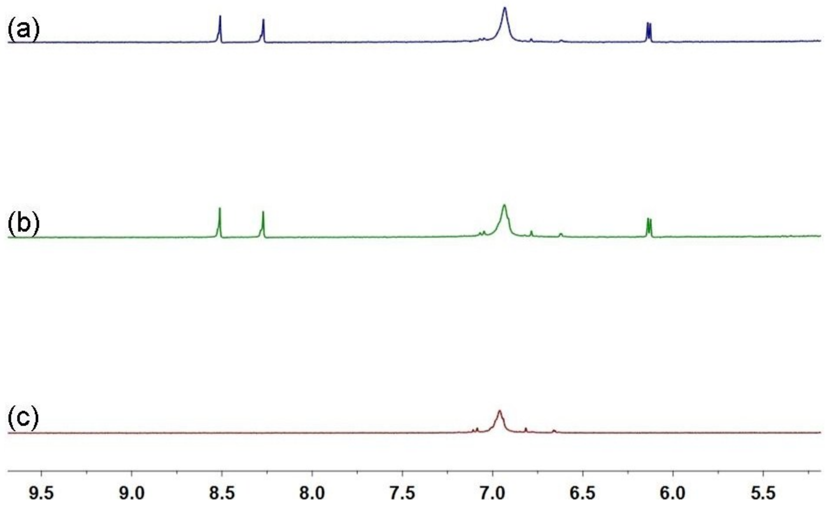

Fig. S14 Partial ^1H NMR spectra (400 MHz, D_2O, 295 K): (a) AMP (1.00 mM); (b) **WP6** (1.00 mM) and AMP (1.00 mM); (c) **WP6** (1.00 mM).

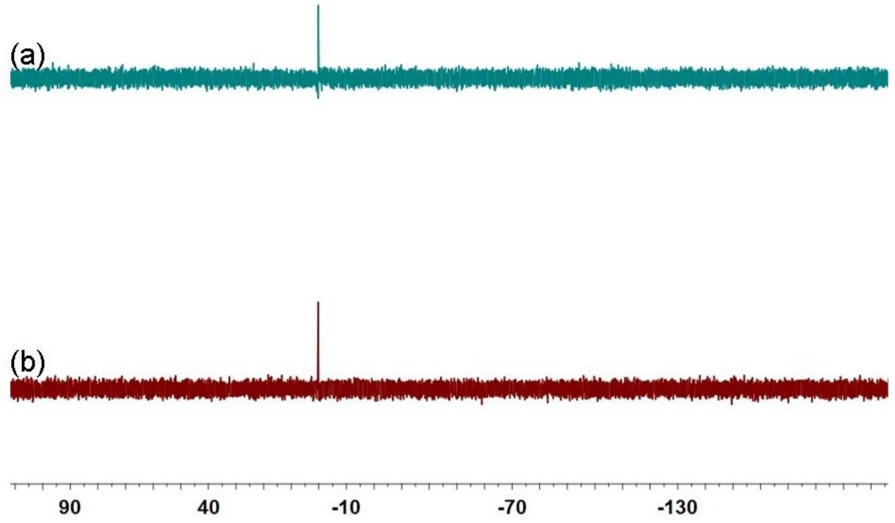

Fig. S15 ^{31}P NMR spectra of (a) AMP (1.00 mM), (b) **WP6** (1.00 mM) and AMP (1.00 mM).

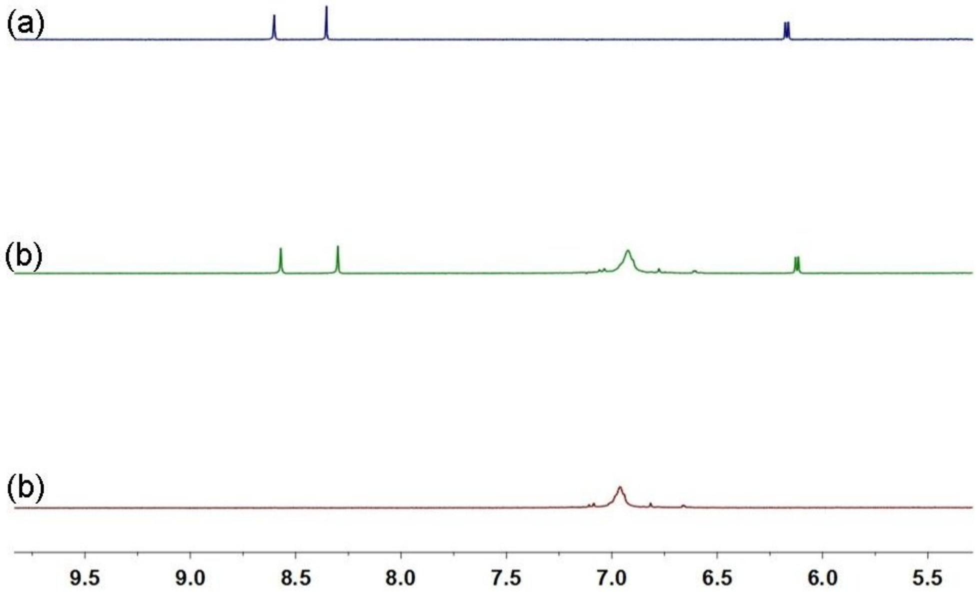

Fig. S16 Partial ^{1}H NMR spectra (400 MHz, D_2O, 295 K): (a) ADP (1.00 mM); (b) **WP6** (1.00 mM) and ADP (1.00 mM); (c) **WP6** (1.00 mM).

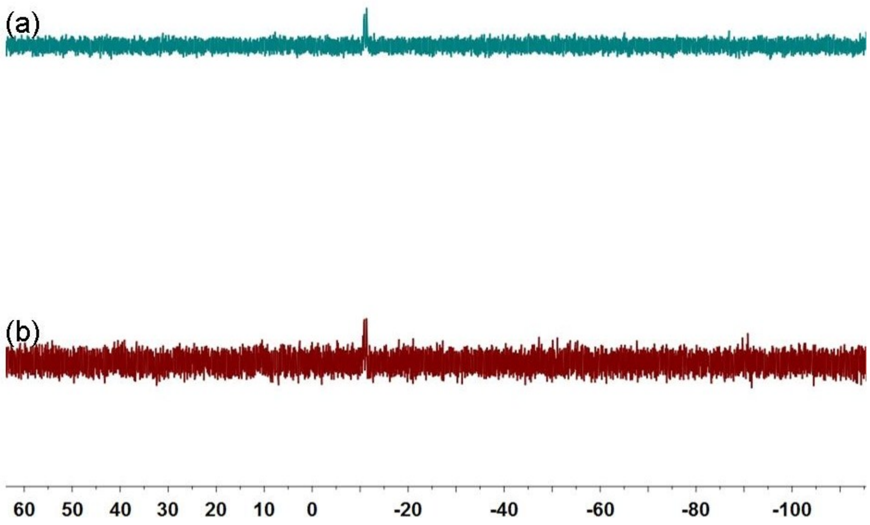

Fig. S17 ^{31}P NMR spectra of (a) ADP (1.00 mM), (b) **WP6** (1.00 mM) and ADP (1.00 mM).

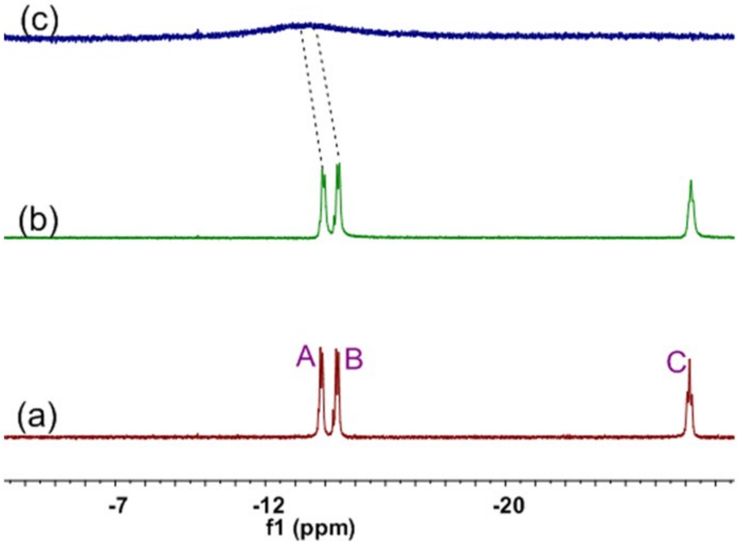

Fig. S18 ^{31}P NMR spectra of (a) ATP (2.00 mM), (b) **WP5** (2.00 mM) and ATP (2.00 mM), and (c) **WP6** (2.00 mM) and ATP (2.00 mM). The phosphorus signals in (a) are assigned as follows:[S2] A. γ-ATP; B. α-ATP; C. β-ATP.

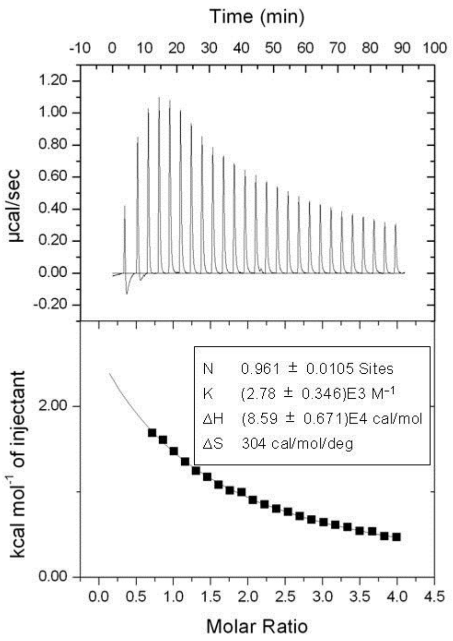

Fig. S19 Microcalorimetric titration of AMP with **WP5** in PBS (pH = 7.4) at 298.15 K. (Top) Raw ITC data for 29 sequential injections (10 μL per injection) of an AMP solution (2.00 mM) into a **WP5** solution (0.100 mM). (Bottom) Net reaction heat obtained from the integration of the calorimetric traces.

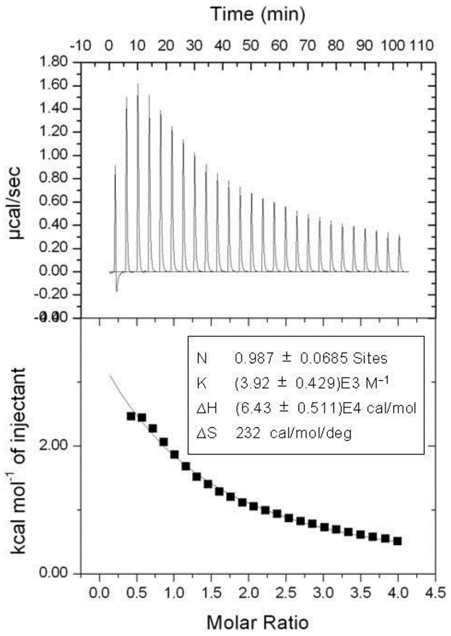

Fig. S20 Microcalorimetric titration of ADP with **WP5** in PBS (pH = 7.4) at 298.15 K. (Top) Raw ITC data for 29 sequential injections (10 μL per injection) of an ADP solution (2.00 mM) into a **WP5** solution (0.100 mM). (Bottom) Net reaction heat obtained from the integration of the calorimetric traces.

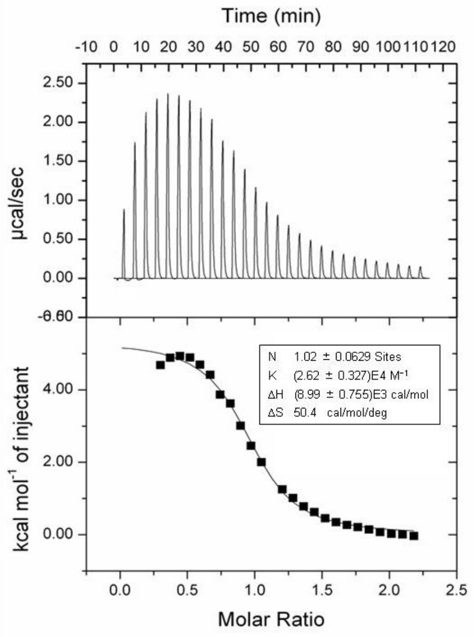

Fig. S21 Microcalorimetric titration of ATP with **WP5** in PBS (pH = 7.4) at 298.15 K. (Top) Raw ITC data for 29 sequential injections (10 μL per injection) of an ATP solution (2.00 mM) into a **WP5** solution (0.100 mM). (Bottom) Net reaction heat obtained from the integration of the calorimetric traces.

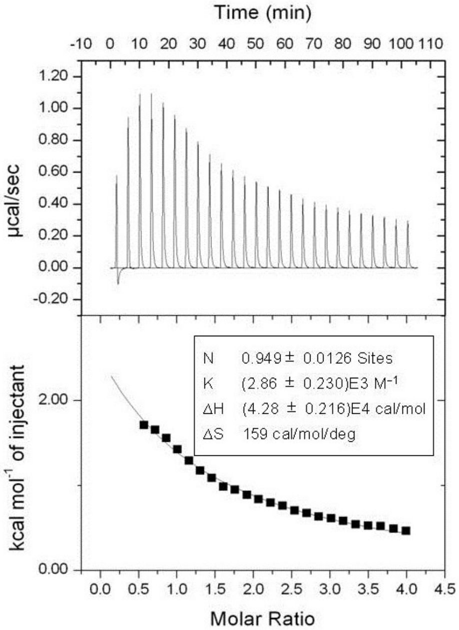

Fig. S22 Microcalorimetric titration of AMP with **WP6** in PBS (pH = 7.4) at 298.15 K. (Top) Raw ITC data for 29 sequential injections (10 μL per injection) of an AMP solution (2.00 mM) into a **WP6** solution (0.100 mM). (Bottom) Net reaction heat obtained from the integration of the calorimetric traces.

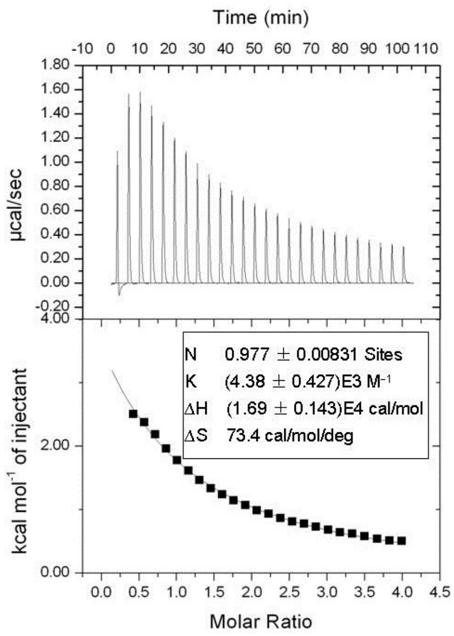

Fig. S23 Microcalorimetric titration of ADP with **WP6** in PBS (pH = 7.4) at 298.15 K. (Top) Raw ITC data for 29 sequential injections (10 μL per injection) of an ADP solution (2.00 mM) into a **WP6** solution (0.100 mM). (Bottom) Net reaction heat obtained from the integration of the calorimetric traces.

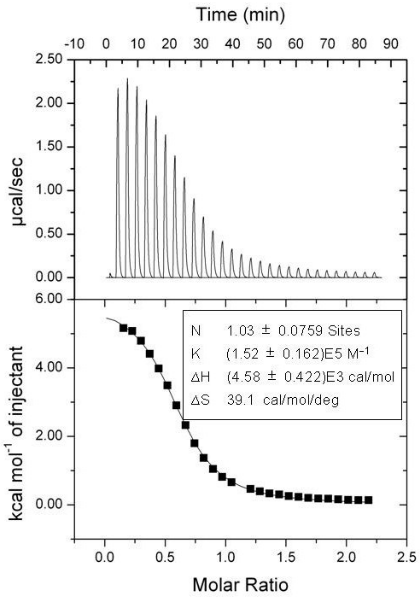

Fig. S24 Microcalorimetric titration of ATP with **WP6** in PBS (pH = 7.4) at 298.15 K. (Top) Raw ITC data for 29 sequential injections (10 μL per injection) of an ATP solution (2.00 mM) into a **WP6** solution (0.100 mM). (Bottom) Net reaction heat obtained from the integration of the calorimetric traces.

From ITC investigations, the enthalpy and entropy changes were obtained ($\Delta H° > 0$; $T\Delta S° > 0$; $|\Delta H°| < |T\Delta S°|$), which indicated that the complexations were driven by entropy changes. The reason is that the "high-energy" water molecules encapsulated in the cavity of **WP5** (or **WP6)** are released to the aqueous bulk by the formation of inclusion hosteguest complexes, which are favorable for entropic gain.[S2]

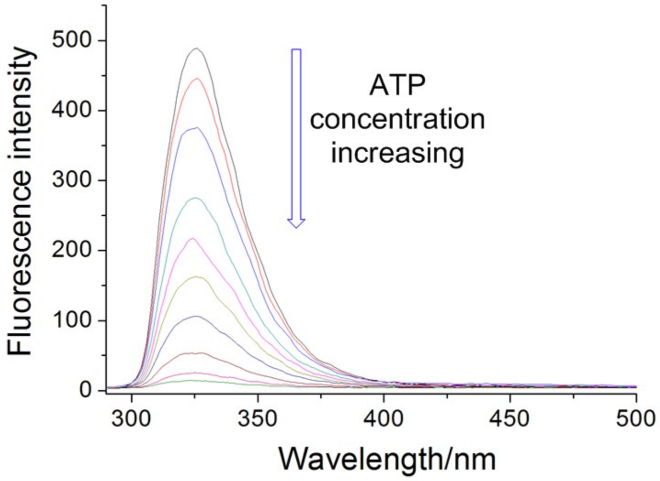

Fig. S25 Fluorescence spectra of **WP6** (2.00×10^{-6} M) in a phosphate buffer solution at room temperature with different concentrations of **ATP** from 0 to 5 equiv.

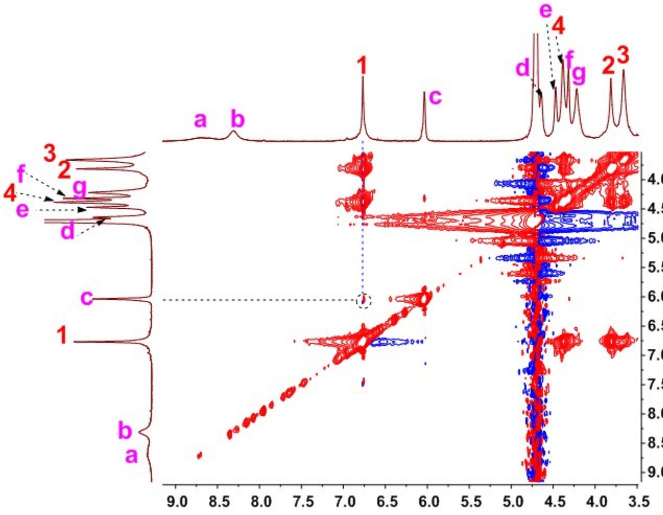

Fig. S26 2D ^1H–^1H NOESY spectrum of **WP6**⊃ATP (400 MHz, D_2O, room temperature). [**WP6**] = 10.0 mM. [ATP] = 50.0 mM.

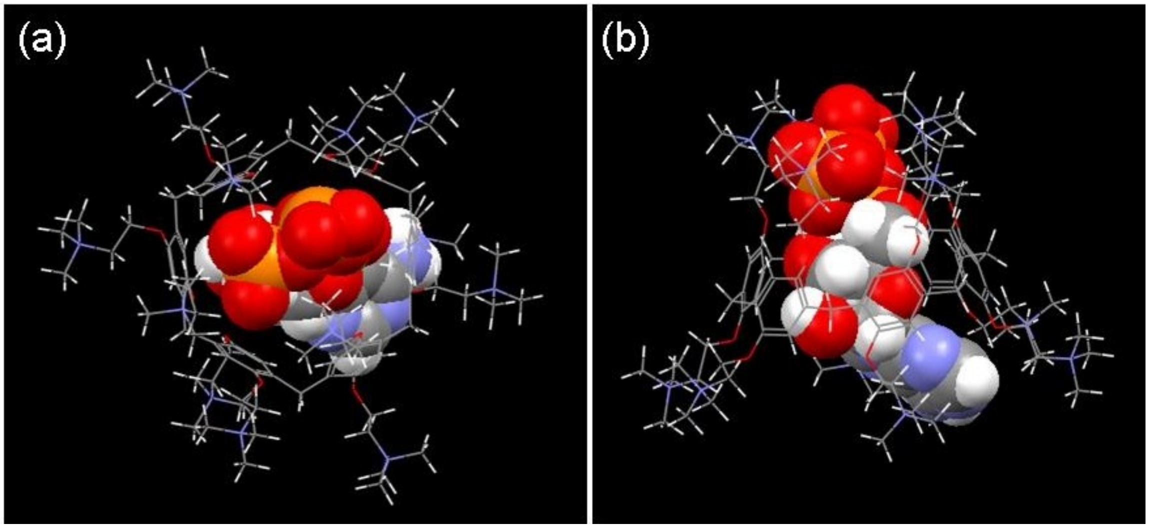

Fig. S27 Energy-minimized structure of **WP6**⊃ATP: (b) top view; (c) side view.

4. Host-induced inhibition of ATP hydrolysis

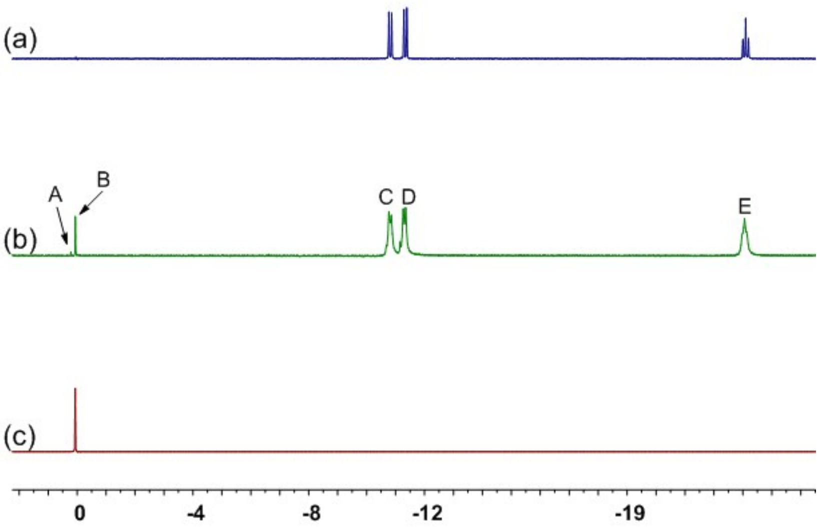

Fig. S28 ^{31}P NMR spectra: (a) ATP (2.00 mM); (b) ATP (2.00 mM) in the presence of alkaline phosphatase (CIAP) (6 U/mL) for 2 h; (c) mM) and ATP (2.00 mM) in the presence of CIAP (6 U/mL) for 12 h. The phosphorus signals in (b) are assigned as follows:[S3] A. α-AMP; B. phosphate; C. γ-ATP; D. α-ATP; E. β-ATP.

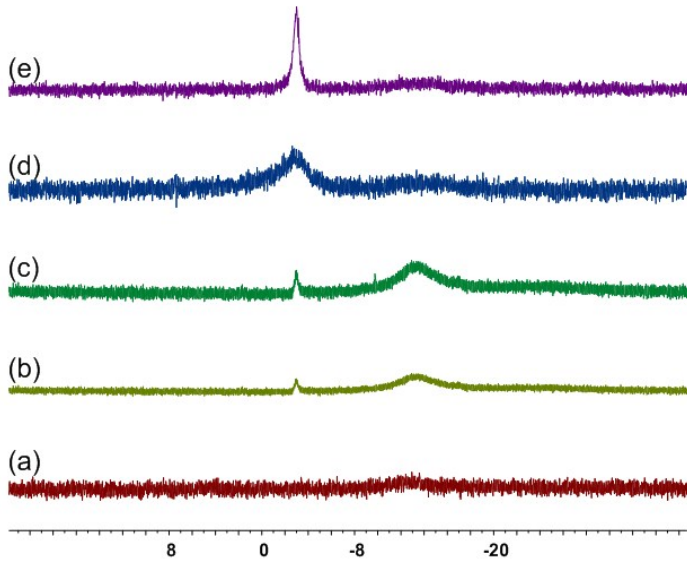

Fig. S29 ^{31}P NMR spectra: (a) **WP6** (2.00 mM) and ATP (2.00 mM); (b) **WP6** (2.00 mM) and ATP (2.00 mM) in the presence of alkaline phosphatase (CIAP) (6 U/mL) for 6 h; (c) **WP6** (2.00 mM) and ATP (2.00 mM) in the presence of CIAP (6 U/mL) for 12 h; (d) **WP6** (2.00 mM) and ATP (2.00 mM) in the presence of CIAP (6 U/mL) for 24 h; (e) **WP6** (2.00 mM) and ATP (2.00 mM) in the presence of CIAP (6 U/mL) for 48 h.

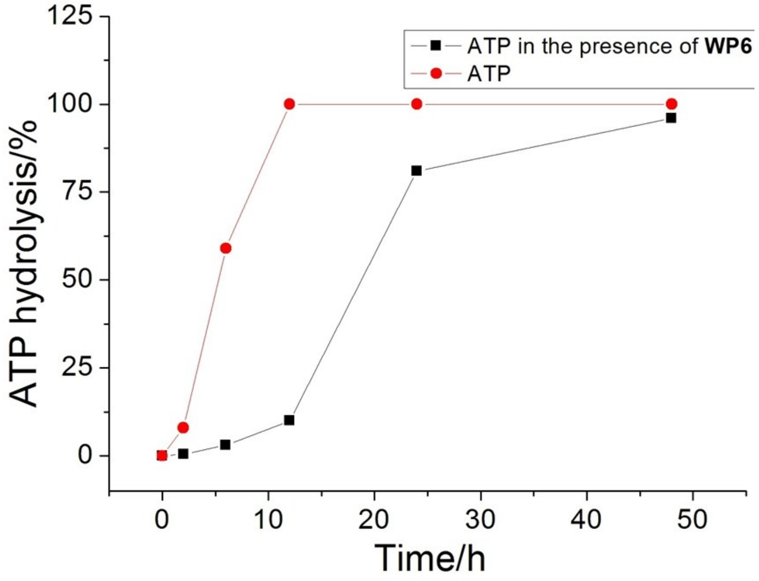

Fig. S30 The hydrolysis of ATP (2.00 mM) in the absence and presence of **WP6** treated with alkaline phosphatase (CIAP) (6 U/mL).

5. Characterizations of PIC micelles

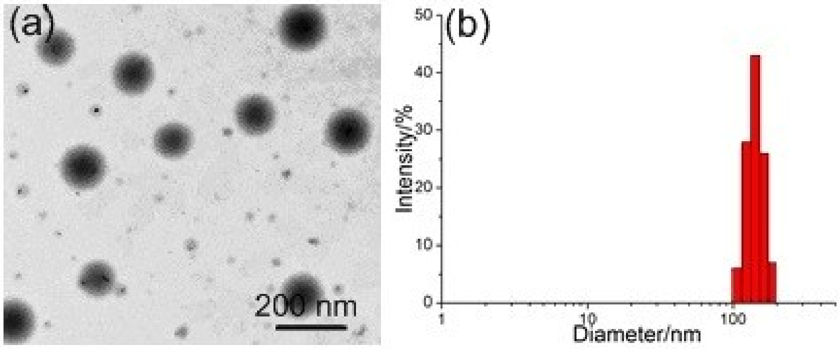

Fig. S31 (a) TEM image and (b) DLS data of DOX·HCl loaded **WP6**/**FA-PEG-*b*-PAA** ternary PIC micelles.

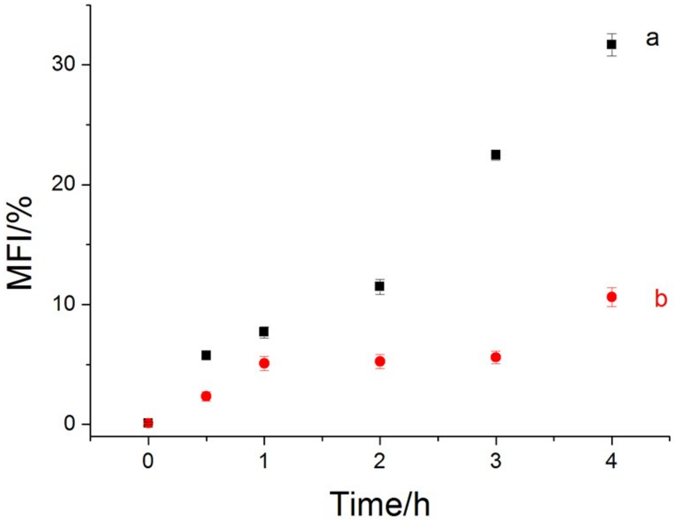

Fig. S32 Mean fluorescence intensity of (a) KB and (b) A549 cells after incubation with culture with DOX·HCl (5.00 μg/mL) loaded **WP6**/**FA-PEG-*b*-PAA** ternary PIC micelles for 0.5 h, 1 h, 2 h, 3 h, and 4 h, respectively.

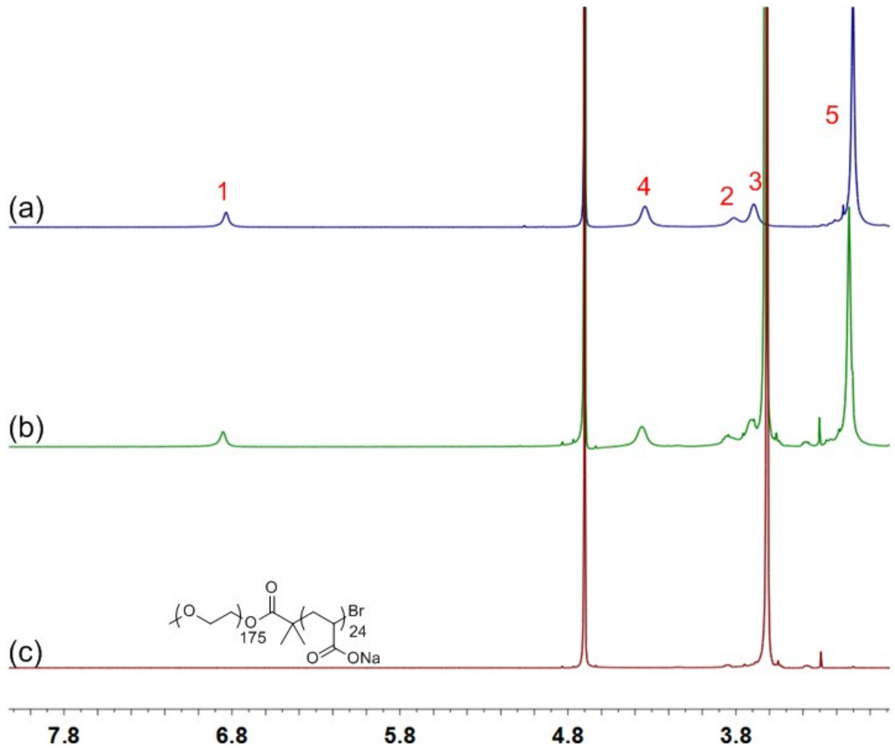

Fig. S33 Partial ^{1}H NMR spectra (400 MHz, D_2O, 295 K): (a) **WP6** (1.00 mM); (b) **WP6** (1.00 mM) and **mPEG-PAANa** (1.00 mM); (c) **mPEG-PAANa** (1.00 mM).

References:

S1. Z. Luo, X. Ding, Y. Hu, S. Wu, Y. Xiang, Y. Zeng, B. Zhang, H. Yan, H. Zhang, L. Zhu, J. Liu, J. Li, K. Cai and Y. Zhao, *ACS Nano*, 2013, **7**, 10271.

S2. F. Biedermann, V. D. Uzunova, O. A. Scherman, W. M. Nau and A. D. Simone, *J. Am. Chem. Soc.*, 2013, **135**, 14879.

S3. Y. Kang, C. Wang, K. Liu, Z. Wang and X. Zhang, *Langmuir*, 2012, **28**, 14562.
